# Structural, Optical, and Sensing Properties of Nb-Doped ITO Thin Films Deposited by the Sol–Gel Method

**DOI:** 10.3390/gels8110717

**Published:** 2022-11-07

**Authors:** Madalina Nicolescu, Daiana Mitrea, Cristian Hornoiu, Silviu Preda, Hermine Stroescu, Mihai Anastasescu, Jose Maria Calderon-Moreno, Luminita Predoana, Valentin Serban Teodorescu, Valentin-Adrian Maraloiu, Maria Zaharescu, Mariuca Gartner

**Affiliations:** 1 “Ilie Murgulescu” Institute of Physical Chemistry, Romanian Academy, 202 Splaiul Independentei, 060021 Bucharest, Romania; 2 National Institute of Materials Physics, 405 bis Atomistilor Street, 077125 Magurele-Ilfov, Romania; 3 Academy of Romanian Scientists, 3 Ilfov Street, 050045 Bucharest, Romania

**Keywords:** Nb-doped ITO thin films, Sol–gel, Optical properties, CO detection

## Abstract

The aim of the present study was the development of Nb-doped ITO thin films for carbon monoxide (CO) sensing applications. The detection of CO is imperious because of its high toxicity, with long-term exposure having a negative impact on human health. Using a feasible sol–gel method, the doped ITO thin films were prepared at room temperature and deposited onto various substrates (Si, SiO_2_/glass, and glass). The structural, morphological, and optical characterization was performed by the following techniques: X-ray diffractometry (XRD), atomic force microscopy (AFM), scanning electron microscopy (SEM), transmission electron microscopy (TEM), and UV/Vis/NIR spectroscopic ellipsometry (SE). The analysis revealed a crystalline structure and a low surface roughness of the doped ITO-based thin films. XTEM analysis (cross-sectional transmission electron microscopy) showed that the film has crystallites of the order of 5–10 nm and relatively large pores (around 3–5 nm in diameter). A transmittance value of 80% in the visible region and an optical band-gap energy of around 3.7 eV were found for dip-coated ITO/Nb films on SiO_2_/glass and glass supports. The EDX measurements proved the presence of Nb in the ITO film in a molar ratio of 3.7%, close to the intended one (4%). Gas testing measurements were carried out on the ITO undoped and doped thin films deposited on glass substrate. The presence of Nb in the ITO matrix increases the electrical signal and the sensitivity to CO detection, leading to the highest response for 2000 ppm CO concentration at working temperature of 300 °C.

## 1. Introduction

Indium tin oxide (ITO) is an n-type semiconductor with a wide energy band gap (3.7 eV), low electrical resistance, and high optical transparency in the visible domain. The development of ITO thin films is of a great interest in the scientific community as a result of their interesting properties, which make them possible candidates for different applications, such as optoelectronic devices [1,2,3], transparent conductive oxides [4], solar cells [5,6,7], gas sensors [8,9], biosensors [10,11,12], thermoelectric applications [13,14], and so on. ITO thin films can be prepared by various physical (magnetron sputtering [15,16,17,18,19], pulsed lased deposition [20], ion beam sputtering [21], and electron beam evaporation [22]) and chemical methods (sol–gel method [23,24], spray pyrolysis [25], and low-temperature combustion synthesis method [26]).

In terms of the gas sensing properties, these materials based on doped or undoped ITO thin films have been proven to detect formaldehyde [27], CO_2_ [28,29,30], CO [31,32,33], NO_2_ [34,35], chlorine [35], benzene [36], toluene [37], and ammonia gases [38,39,40]. Additionally, ITO thin films can sense ethanol [41,42] and water vapors [43]. Furthermore, ITO-coated glass substrates were used in liquid crystals display (LCD) [44,45,46,47] and polymer dispersed liquid crystals device fabrication [48,49,50]. The suitable material for a certain application can be achieved by changing several parameters involved in the preparation process as follows: the deposition technique [16], the metal doping level [51], the pre- and final annealing temperatures [19], and the film thickness [22].

Carbon monoxide (CO) is among the most harmful gases, being associated with several health problems, even death depending on exposure time. CO is released into the environment because of the partial combustion of fuels from cars and domestic or industrial activities. Consequently, CO detection is necessary because of its odorless and colorless properties [52,53]. Over time, researchers have studied metal-oxides based systems, such as SnO_2_, In_2_O_3_, ZnO [8,54], and indium-tin oxide (ITO) [55,56], with the aim to develop CO sensors. According to scientific reports, the properties of ITO thin films can be tailored by doping with various metals: Ag [57], Ga [58], Cr [59], Zn [30,60], Ti [61], Nb [62], and so on. In contrast with metal-doped ITO films, there are few reports concerning Nb-doped ITO thin films [51,62,63,64]. Therefore, these films were successfully obtained by radio frequency (RF) sputtering, pulsed laser deposition (PLD), and sol–gel methods to investigate the transparent conductive oxide, optoelectronic, and electrochromic properties [51,62,63,64]. The sol–gel method is a versatile and efficient procedure for the preparation of pure and doped metal oxide films or powders [65,66], showing some advantages such as purity, homogeneity, possibility to introduce dopants in large quantities, low processing temperature, ease of manufacturing, control over the stoichiometry, composition, and viscosity [67].

In this work, we explored the structural, morphological, and optical properties of multilayer Nb-doped ITO thin films prepared by the sol–gel method, deposited onto different substrates (glass, SiO_2_/glass, and Si). The effects of the dopant (4% Nb) and of the type of substrate were examined by X-ray diffraction (XRD), atomic force microscopy (AFM), scanning electron microscopy (SEM), transmission electron microscopy (TEM), and spectroscopic ellipsometry (SE). The Nb doping level was chosen based on our previous work regarding the Zn-doped ITO as a CO_2_ sensor.

As aforementioned, our aim was also to examine the influence of Nb doping on the detection properties of ITO thin films. Although this type of material was previously studied for certain applications, as far as the authors are aware, there are no literature data regarding its sensing properties. Therefore, the novelty of this work was the improvement in the electrical properties of ITO film through Nb doping, making it a more sensitive material for CO detection. Accordingly, the sample with the most suitable properties (thickness and porosity) was used for gas measurements of CO.

## 2. Results and Discussion

### 2.1. Structural Characterization

#### XRD Analysis

**Figure 1:**
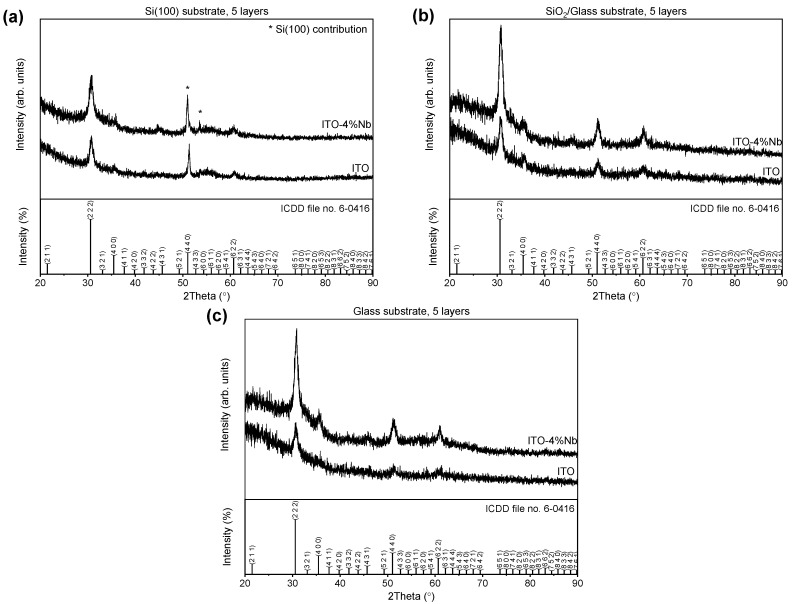
XRD patterns of ITO films undoped and doped with 4% Nb deposited on (**a**) Si, (**b**) SiO_2_/glass, and (**c**) glass.

Figure 1a–c shows the XRD profiles of multilayer ITO/Nb thin films. Different substrates (Si, SiO_2_/glass, and glass) were coated by five successive layers using the ITO/Nb sol–gel solution. To highlight the influence of the dopant on the ITO film structure, previously reported data [30] on the undoped ITO thin films are presented in Figure 1. The diffraction lines, corresponding to crystal planes (2 2 2), (4 0 0), (4 4 0), and (6 2 2), were observed for both ITO/Nb and ITO thin films. ITO, which is Sn-doped In_2_O_3_, crystalizes in the bixbyite-type cubic structure of In_2_O_3_, with Ia-3 space group (ICDD file no. 06-0416). Except for the diffraction line of Si (marked with an asterisk on the Figure 1a) belonging to the substrate, no other phases were detected in the XRD patterns, indicating that the Nb and Sn dopants were incorporated into the In_2_O_3_ structure. The doped samples present an improved crystallinity, based on the shape of the diffraction line (higher intensity and narrower width). The lattice constants are slightly larger for the doped samples, most likely owing to the incorporation of the dopants into the cubic bixbyite structure. The crystallite size was estimated using Scherrer’s equation [68] only for the crystal plane (222) and was found to be around 10 nm (Table 1).

(1) $$D=\frac{0.94\times \lambda }{\beta \times cos\theta },$$

where *D* is the average size of the crystallites, *λ* is the X-ray wavelength, *β* is the full width at half the maximum intensity (FWHM), and *θ* is the location of the diffraction line (Bragg angle).

The ITO/Nb films, as well as undoped ITO films, deposited on the SiO_2_/glass type of support show a better crystallinity in relation to those deposited on glass or Si substrate.

### 2.2. Morphological Studies

#### 2.2.1. AFM Measurements

**Figure 2:**
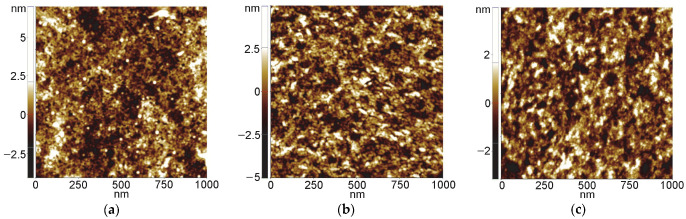
Topographic 2D AFM images scanned over an area of (1 µm × 1 µm), showing the morphology of the ITO/Nb films deposited on (**a**) glass, (**b**) SiO_2_/glass, and (**c**) Si.

**Figure 3:**
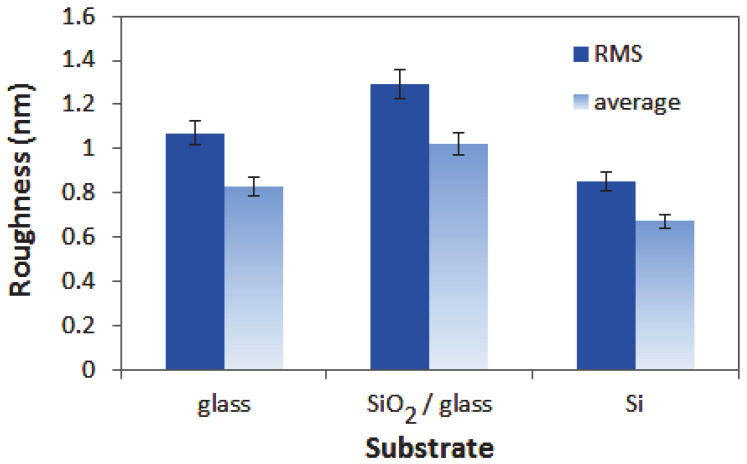
RMS (solid fill) and average (gradient fill) roughness for the ITO/Nb films deposited on different substrates.

The surface morphology and the roughness of the ITO/Nb thin films were assessed by AFM. Figure 2 shows the 2D topographic AFM micrographs at the scale of (1 × 1) μm^2^ for the ITO/Nb films deposited on three different substrates: glass (Figure 2a), SiO_2_/glass (Figure 2b), and Si (Figure 2c). As could be seen, the films are compact and exhibit a uniform structure of nanometric-sized particles (Figure 2) with the root-mean-square (RMS) roughness values in the range of 0.85–1.29 nm and average roughness in the range of 0.67–1.02 nm (Figure 3). It is suggested that the sol–gel deposition of a SiO_2_ layer between the glass substrate and the ITO/Nb film slightly increases the roughness of the ITO film, in comparison with the ITO film deposited directly on glass, related to a better crystallinity as observed in XRD. On the other hand, the deposition of the ITO film on Si leads to the lowest roughness in this series, indicating a denser layer (in agreement with the refractive index curves).

#### 2.2.2. SEM Investigation

**Figure 4:**
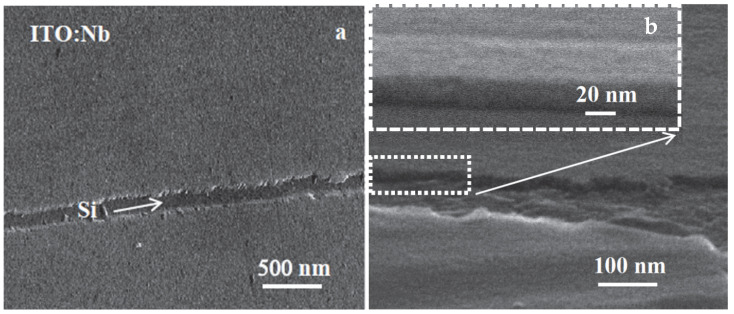
SEM micrographs at different magnification: (**a**) 20,000× and (b) 100,000× of the tilted ITO/Nb film deposited on Si, showing the smooth film surface and the film thickness.

SEM was also used for the characterization of ITO/Nb films. As can be seen in the tilted film micrographs of different magnifications (Figure 4), the surface of the film is very smooth. Figure 4a is a low-magnification (20,000×) micrograph showing a scratch on the film surface, where the Si substrate (the darker zone marked with an arrow) is exposed. Figure 4b is a higher-magnification image (100,000×) showing the step at the edge of the film on top of the Si substrate, indicating that the film thickness is above 20 nm. The inset is the magnification of the area marked with a square in Figure 4b.

The elemental compositional analysis by energy dispersive spectroscopy (EDX) (Table 2) revealed that niobium is incorporated into the film. A cationic ratio Nb/(In+Sn) of 0.037 was determined from the EDX measurements for the Nb-doped film.

#### 2.2.3. TEM Analysis

As revealed by TEM images, the thickness of the ITO/Nb film deposited on Si is generally between 26 and 29 nm for the main layer, which is in accordance with SEM and SE results.

**Figure 5:**
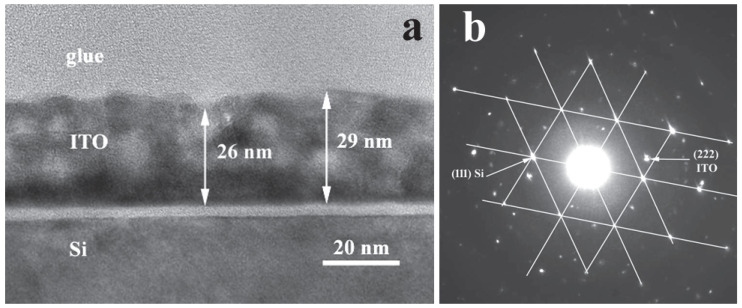
(**a**) Low-magnification XTEM image of the ITO/Nb film on Si and (**b**) its SAED pattern.

The low-magnification XTEM images (Figure 5a) show that the film has crystallites of the order of 5–10 nm, as well as relatively large pores, around 3 to 5 nm in diameter. The deposited layers are not clearly distinguishable; instead, there is an apparent morphology in three layers separated by rows of pores (Figure 5a).

**Figure 6:**
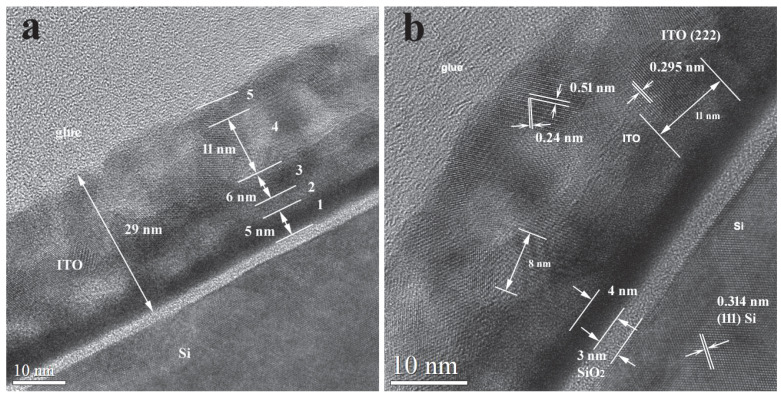
HRTEM images of the ITO:Nb film on Si: (**a**) morphology of the layers and (**b**) crystallite size and coherent lattice fringes.

The high-resolution transmission electron microscopy (HRTEM) images of the ITO/Nb film on Si (Figure 6a) exhibit that the first layer is denser (1), followed by a pore area layer (2) and then another denser layer (3) of half thickness. In the upper area (4), no layers can be distinguished, but there is a mixture of crystallites and pores. The surface layer (5) looks like a “crust” and is less compact. No pores are present in this upper layer, leading to low roughness values as observed in AFM. The polycrystalline ITO film structure has no texture, as revealed by the SAED pattern exposed in Figure 5b. The Si substrate is oriented along the [1 1 0] zone axis and the Si reflections are connected by the white line in the SAED pattern. The HRTEM obtained in the thinner area of the XTEM specimen (Figure 6b) shows a dense morphology of the layer at the bottom of the film. This layer is from 4 to 5 nm thick and can be probably identified with the real first deposited layer. In the rest of the film, the pores appear in the film volume. These pores are aligned in the bottom part of the film but are randomly arranged in the rest of the film. The pores are not clearly delimited and can also consist of less dense zones containing some amorphous material. The coherent lattice fringes in the HRTEM images (Figure 6b) revealed that the ITO crystallites are in the range of 5 and 10 nm. The morphology of the layer-by-layer deposited ITO film is strongly affected by the crystal growth process, because the final size of the ITO crystallites is bigger than the initial thickness of each deposited layer (about 5 nm). If we compare these results with the case of Zn-doped ITO films [30], it can be observed that the crystallization process and the total film thickness are influenced by the dopant nature [30].

The morphology of the apparent layers is shown in Figure 6a. The ITO crystallites size is revealed in the image by the coherent lattice fringes areas (Figure 6b). At the interface of the ITO film with the Si substrate, a SiO_2_ layer with a thickness of about 3 nm is formed.

### 2.3. Optical Characterization

#### SE in UV/Vis/NIR Domain

**Figure 7:**
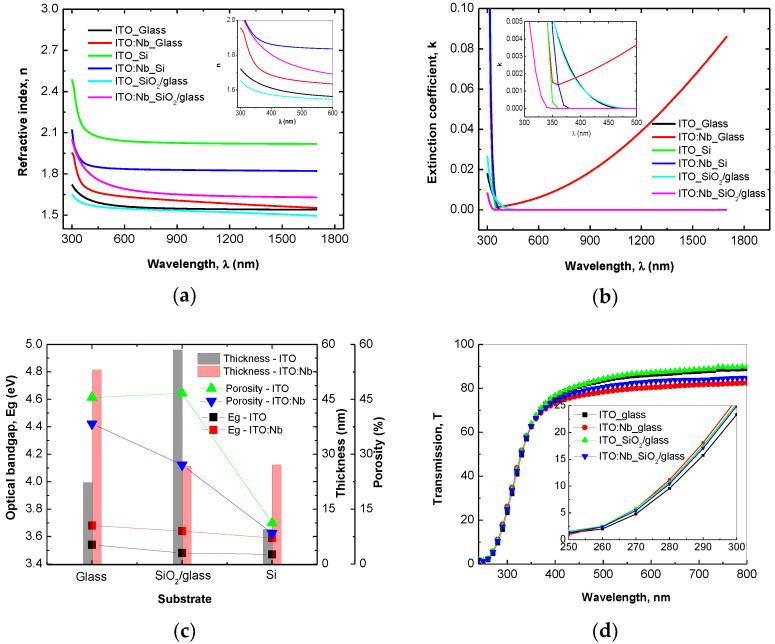
Optical constants (**a**) n, (**b**) k, (**c**) optical band gap—Eg, thickness, porosity—P, and (**d**) transmission—T of undoped and doped ITO thin films.

In the UV/Vis/NIR ellipsometric data analysis, the “General Oscillator” model [69] was applied to the ITO/Nb structure considering Tauc–Lorentz and Drude oscillators. The surface roughness was considered a mixture of 50% material (film) and 50% voids (air) and was fitted with the Bruggeman’s effective medium approximation (B-EMA) [70]. The layer thicknesses (d_film_), the optical constants (refractive index—n and extinction coefficient—k), and the band gap energy (Eg) computed by Tauc formula [71] of the ITO/Nb films evaluated from the best fit are presented in Figure 7 and Table 3. A regression analysis of optical data, based on MSE, was used to evaluate the fit quality [69].

The porosity (P) of the films was calculated with the following formula [72]:

(2) $$P=\left[1-\frac{n^{2}-1}{n_{d}^{2}-1}\right]\times 100\;\left(\%\right),$$

where *n_d_* = 1.92 is the refractive index of the pore-free ITO (at λ = 500 nm) from WASE program and n is the refractive index of the ITO/Nb film at the same wavelength (Figure 7c).

The ITO/Nb films deposited on Si (8.44%) have the lowest porosity compared with those deposited on SiO_2_/glass (27.09%) and glass (38.32%) (see Table 3). The transmission spectra (T) of the ITO/Nb films measured in the 250–900 nm spectral range are shown in Figure 7 and their values at λ = 500 nm are indicated in Table 3. The ITO/Nb films deposited on glass and SiO_2_/glass exhibit a good transmittance (~80%) from visible and increase to 85% at 800 nm. It was observed that the Nb-doping of ITO reduces the band gap of thin films and can be attributed to the Burstein–Moss shift [73] in the visible domain (Figure 7c).

### 2.4. Gas Sensing Measurements

#### CO Sensing Measurements

Both the comprehension of the gas/solid interaction mechanism and identification of active regions in the films (surface, grain, and grain boundaries), which are implied in analyzed gas sensing, were assessed through complex impedance analysis. Using Nyquist plots (Z” vs. Z’), the impedance measurement results (Z = Z’ + j Z′′, where Z’ and Z” were the real and imaginary components, respectively) were represented.

Upon the exposure to a reducing gas, the resistance of the undoped and ITO/Nb films deposited on glass decreased, while the exposure to air led to an increase in this parameter. From our gas measurement results, it was concluded that the investigated films exhibited an n-type conductivity, as a consequence of the changes in terms of resistance of the films, function of the reducing gas, or air exposure.

**Figure 8:**
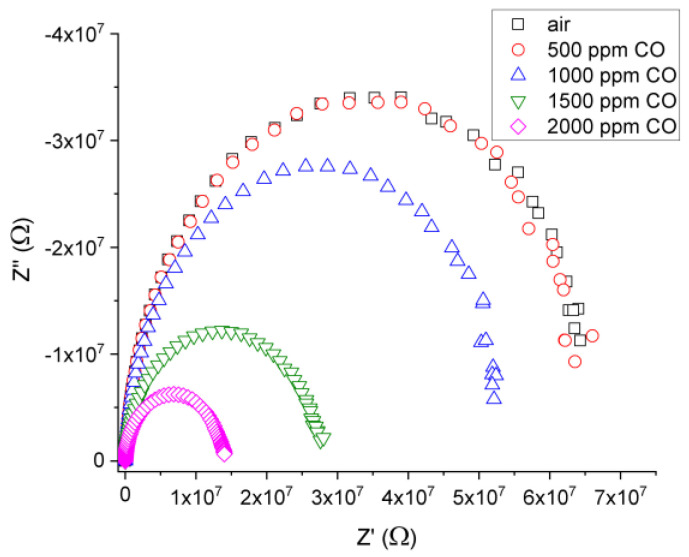
Nyquist plots for the ITO/Nb glass film at 300 °C for different CO concentrations.

**Figure 9:**
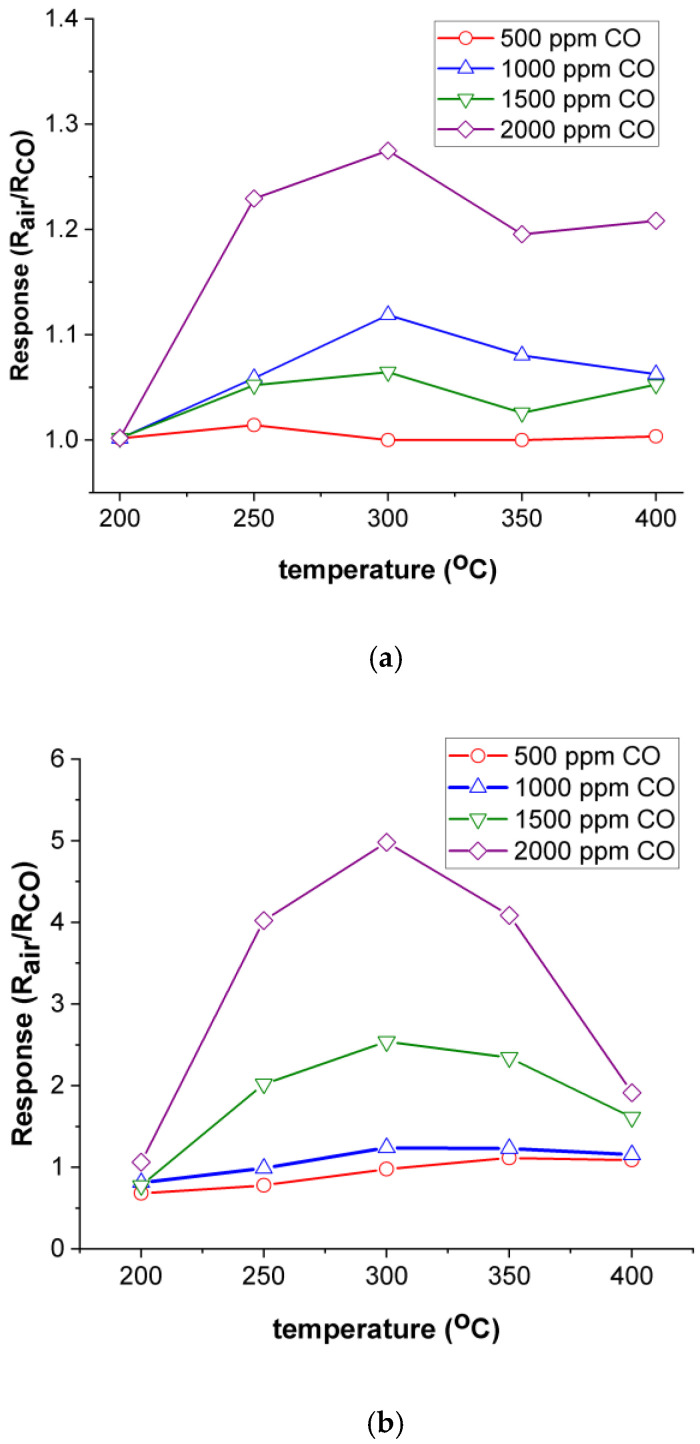
Electrical response of (**a**) ITO/Nb glass and (**b**) ITO/glass film for various concentrations of CO versus the working temperature.

From the intersection of the semicircle in Nyquist plots (Z” vs. Z’), we can determine DC-resistance for our films. In Figure 8, Nyquist plots are presented for ITO/Nb glass at 300 °C for different CO concentrations. In Figure 9, the electrical response of ITO/Nb glass and ITO glass film is plotted for various concentrations of CO function of the working temperature. The difference in sensitivity between the two samples can be associated with the presence of Nb in the ITO film.

As observed, the response of the Nb-doped ITO/glass sample exhibits a maximum at 300 °C (R_air_/R_CO_ = 5), which will be considered the optimum working temperature of the material. It can also be stated that the films are most sensitive to the 2000 ppm CO concentration. The maximum sensitivity of ITO/Nb glass is approximately four times higher than the data achieved for Nb-doped TiO_2_ samples deposited in similar conditions through a sol–gel approach [74].

## 3. Conclusions

ITO/Nb thin films were successfully deposited onto three different substrates through the sol–gel method. XRD analysis proved the polycrystalline nature of the films. AFM measurements indicated that all ITO/Nb films exhibit low surface roughness values, below 1 nm. SEM investigation revealed that the films are continuous, homogeneous, and adherent to the substrate. TEM analysis showed that the ITO/Nb films are very thin (26–29 nm), in agreement with SEM and SE, but with a complex morphology (a detailed study will follow in a next paper). The low-magnification XTEM images show that the film has crystallites of the order of 5–10 nm, as well as relatively large pores, around 3 to 5 nm in diameter, as also seen in the coherent lattice fringes of the HRTEM. The polycrystalline ITO film structure has no texture, as observed in SAED patterns. The morphology of the layer-by-layer deposited ITO film is significantly affected by the crystal growth process, because the final size of the ITO crystallites is bigger than the initial thickness (~5 nm) of each deposited layer. The Nb doping of ITO reduced the band gap of the films and can be attributed to the Burstein–Moss shift in the visible domain. The optical transmittance of the films deposited on transparent substrates (glass and SiO_2_/glass) was found to exceed 80%. The detection properties were characterized in terms of resistance and gas-sensing response. It was found that the response of the ITO/Nb glass sample exhibits a maximum at 300 °C (R_air_/R_CO_ = 5) and that the ITO films are most sensitive to the 2000 ppm CO concentration. The sensitivity data are promising, but still preliminary, and will be expanded in future studies.

## 4. Materials and Methods

### 4.1. Thin Film Deposition

**Figure 10:**
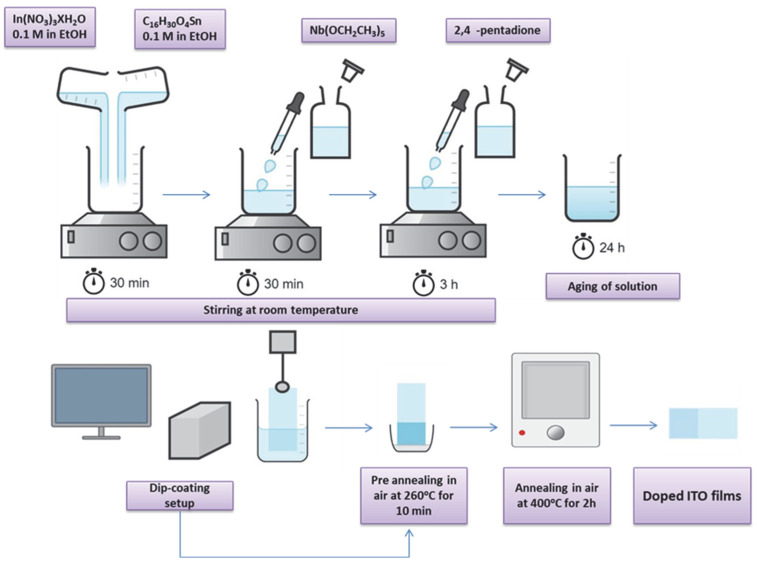
The flow chart of the ITO/Nb thin films preparation.

The Nb-doped ITO (ITO/Nb) films were prepared by the sol–gel method on the investigated substrates (glass, SiO_2_/glass, and Si) using the following as precursors: indium nitrate and 2-tin-ethyl hexanoate as In_2_O_3_ and SnO_2_ sources, 2,4-pentanedione as chelating agent, and niobium (V) ethoxide as dopant. Figure 10 describes the procedure for ITO thin films preparation: In(NO_3_)_3_·H_2_O and 2-tin-ethyl hexanoate solutions of 0.1 M concentration were homogenized by magnetic stirring at room temperature, obtaining a clear transparent solution. The dopant precursor (niobium (V) ethoxide) was added in the solution after 30 min of homogenization. Acetyl-acetone was added after 30 min and the homogenization continued for 3 h at room temperature. A light-yellow sol was obtained and it was kept at room temperature for 24 h. The as-obtained sol was used for the thin film deposition. The obtaining of ITO/Nb films with five layers was carried out by repetitive depositions (at a 5 cm/min withdrawal rate). The final films were achieved after 2 h of annealing treatment at 400 °C, with the heating rate of 5 °C/min. For the SiO_2_-coated glass substrate (SiO_2_/glass), the SiO_2_ layer was prepared according to the sol–gel method as presented in our previous work [75], preventing the diffusion of some elements from glass to ITO.

### 4.2. Thin Film Characterization

The structure of the ITO/Nb films was evaluated by the X-ray diffraction (XRD) method. XRD patterns were recorded using a Rigaku Ultima IV multifunctional diffraction system (Rigaku Corp., Tokyo, Japan), with Cu Kα (λ = 1.5406 Å) radiation, generated at a voltage of 30 kV and a current of 30 mA. The diffractometer was set in thin film geometry with a fixed incidence angle at α = 0.5°. The measurements were performed at a scan rate of 5° (2θ)/min over a range of 5–90°. Crystallite size was obtained from the Scherrer’s formula only for the crystal plane (222).

AFM measurements were performed with an XE-100 apparatus (Park Systems) selecting the so-called non-contact working mode, in order to decrease the tip–sample interaction. All AFM images were registered using NCLR tips (Nanosensors™), with less than 8 nm radius of curvature. The AFM micrographs were processed with XEI (v.1.8.0) Image Processing Program developed by Park Systems for tilt correction and roughness evaluation.

Microstructural evaluation of the samples was achieved by SEM investigations using a FEI Quanta 3D microscope operating in the range of 5 and 30 kV.

TEM analysis working in low and high resolution as well as selected area electron diffraction (SAED) using a JEOL ARM200F analytical electron microscope operated at 200 kV was performed for systematic morphological investigations of the prepared thin films. The sample was prepared through the classical method of cross section by cutting 2 × 1 mm^2^ pieces, gluing them face to face, followed by mechanical polishing and final ionic thinning with the help of a Gatan PIPS System.

SE measurements were carried out at room temperature on J.A. Woollam Co. Inc. (Lincoln, NE, USA) equipment composed of a variable angle spectroscopic ellipsometer. The SE spectra were recorded in the 300–1700 nm (UV/Vis/NIR) wavelength range with a 10 nm step, at an incident angle of 70°. For multi-parameter fitting, WASE program provided by Woollam was used. To minimize the difference (mean square error—MSE) between the experimental and the theoretical data, an iterative least-squares method was used. From the ellipsometric data analysis, the film thickness and the refractive index (n) were obtained with an accuracy of ±0.2 nm and ±0.005, respectively. The optical transmission was measured with the same equipment at a 0° incidence angle.

The ITO/Nb films deposited on glass were evaluated for gas sensing performances by impedance measurements. The four-point probe method inside a Probostat standard cell was used for gas sensing measurements. The samples were placed in a controlled atmosphere under a continuous gas flow of 177 mL/min, using a calibrated system of mass-flow controllers. Air and CO were mixed inside a vessel placed before the inlet of the impedance measurement cell. The electrical measurements were performed with a four-probe method AC impedance spectrometer equipped with a Solartron 1260 electrochemical interface, with an applied AC bias amplitude of 500 mV. Electrochemical impedance spectra (EIS) were recorded in the frequency domain from 3 MHz to 100 Hz at temperatures of 200 to 400 °C with a ProboStat cell (NorECs, Oslo, Norway).

## Figures and Tables

**Table 1:** Structural parameters and crystallite size of undoped and Nb-doped ITO films deposited by the sol–gel method on three different substrates.

Sample Name	*d*-Value	FWHM	Lattice Constants		Size *D*
			*a = b = c*	*α =β = γ*	
	(Å)	(°)	(Å)	(°)	(nm)
ITO on SiO_2_/glass	2.912(4)	0.77(5)	10.101(10)	90	11
ITO on Si	2.910(4)	0.82(5)	10.106(6)	90	10.5
ITO on Glass	2.907(6)	0.90(7)	10.137(14)	90	9.5
ITO/Nb on SiO_2_/glass	2.910(2)	0.89(2)	10.1219(3)	90	10
ITO/Nb on Si	2.912(4)	1.00(5)	10.1356(6)	90	9
ITO/Nb on Glass	2.907(3)	0.86(3)	10.103(6)	90	10

**Table 2:** Cation composition measured by EDX elemental analysis of the ITO/Nb film deposited on Si substrate.

	In (%)	Sn (%)	Nb (%)
ITO	83.1	16.9	0
ITO/Nb	79.9	16.4	3.7

**Table 3:** Parameters determined by SE analysis of ITO/Nb thin films for different substrates.

Parameters	Glass	SiO_2_/Glass	Si
d_SiO2_ * (nm)	-	40.3	2.4
d_film_ (nm)	53.9	26.7	27.1
d_rough_ * (nm)	1.9	0.1	1.4
MSE	4.07	1.38	1.68
n **	1.63	1.72	1.85
Eg(eV)	3.68	3.64	3.59
T ** (%)	78.45	80.64	-
P ** (%)	38.32	27.09	8.44

* Note: d_SiO2_ is SiO_2_ thickness and d_rough_ is the thickness of the roughness; ** note: n, T, and P are calculated for λ = 500 nm.

## Data Availability

The data presented in this study are available upon request from the corresponding author.
